# Protection induced in pigs previously infected by the non-virulent strain 1330 of ***Streptococcus suis*** serotype 2 is not due to the secretion of the bacteriocin suicin

**DOI:** 10.1371/journal.pone.0323370

**Published:** 2025-05-29

**Authors:** Samantha J. Hau, Nahuel Fittipaldi, Servane Payen, Daniel Grenier, Daniel W. Nielsen, Susan L. Brockmeier, Marcelo Gottschalk

**Affiliations:** 1 Virus and Prion Research Unit, National Animal Disease Center, ARS, USDA, Ames, Iowa, United States of America; 2 Groupe de recherche sur les maladies infectieuses en production animale, Faculté de médecine vétérinaire, Université de Montréal, St-Hyacinthe, QC, Canada; 3 Centre de Recherche en Infectiologie Porcine et Avicole (CRIPA), Fonds de Recherche du Québec - Nature et Technologies (FQRNT), Saint-Hyacinthe, QC, Canada; 4 Groupe de Recherche en Écologie Buccale (GREB), Faculté de Médecine Dentaire, Université Laval, Quebec City, QC, Canada; 5 Ruminant Diseases and Immunology Research Unit, National Animal Disease Center, ARS, USDA, Ames, Iowa, United States of America; University of Messina: Universita degli Studi di Messina, ITALY

## Abstract

*Streptococcus suis* is a systemic pathogen of swine and imposes a significant economic burden on the swine industry. Disease with *S. suis* is controlled with antibiotic treatment and vaccination with inactivated vaccines, which can be derived from the strains circulating on the farm. Inactivated vaccines have shown mixed results with minimal data supporting reductions in morbidity and mortality following their use. With increasing restrictions on antibiotic use and increasing concerns surrounding antimicrobial resistance, alternatives to antibiotics or novel, highly effective vaccines are needed for treating or preventing disease with *S. suis*. Bacteriocins are a potential alternative to antibiotics, as bacteriocins are antimicrobial peptides produced by bacteria. However, the use of bacteriocins to limit pathogenic *S. suis* remains relatively under-examined. Live vaccines are a potential novel and effective method of preventing disease, as they provide competition for pathogenic strains and would limit pathogenic strain colonization while stimulating a protective immune response. This study investigated the use of an avirulent, bacteriocin producing isolate of *S. suis* (90–1330) as an intranasal vaccine and evaluated the role of the bacteriocin by comparing protection to animals inoculated with a mutant lacking bacteriocin production (90–1330Δ*suicin*). Animals were protected from systemic disease when challenged with a virulent isolate 21 days after inoculation with either 90–1330 or 90–1330Δ*suicin* but were not protected when challenged 3 days after inoculation. Evaluation of antibody titers showed increased titers 21 days post-inoculation, and the humoral response was likely providing systemic protection. Although 90–1330 was unable to protect animals challenged 3 days post-inoculation, the strain should be considered a good candidate for vaccine development. *S. suis* 90–1330 was able to induce a protective immune response with a single intranasal inoculation and bacteriocin production may be able to contribute to protection when animals have a lower exposure dose, as in a production setting.

## Introduction

*Streptococcus suis* is a globally disseminated zoonotic pathogen causing significant economic losses in the swine industry [1]. *S. suis* causes severe systemic disease, including meningitis, polyarthritis, and polyserositis mostly in 5- to 10-week-old piglets. Although certain strains of *S. suis* can cause severe disease, *S. suis* is ubiquitous and can be found in the upper respiratory tract of healthy animals without causing disease [1–3]. Genomic analysis has revealed that *S. suis* isolates have a complex population structure and marked differences can be seen between known virulent and avirulent *S. suis* isolates [4]. The mechanisms by which *S. suis* isolates cause disease are only partially understood; however, many putative virulence factors or virulence associated proteins have been reported [3,5,6]. The correlation between individual putative virulence factors and disease has not clearly been established, indicating virulence is multifactorial [3,5,6]. In addition, other concomitant factors may influence the development of disease, such as environmental and management factors like high humidity, poor ventilation, crowding, and commingling of pigs from different sources, as well as host immune status and the presence of coinfections [7].

Prevention of *S. suis* has focused on the control of predisposing factors, nutritional management [8], and the development of broadly protective vaccines. The latter has been complicated by limited understanding of *S. suis* virulence and the genetic variability of *S. suis* isolates. Currently, available vaccines are generated from inactivated bacteria (bacterins) and often derived from the strain circulating on the farm (autogenous vaccines) [9,10]. The efficacy of autogenous vaccines is controversial, and they are specific to an individual farm and to the producing laboratory [10,11]. Current research has focused on development of alternative vaccine platforms, including subunit and vectored vaccines [9,12–15], and other non-antibiotic intervention strategies, such as bacteriocins and bacteriophages or their lysins [16–20].

Bacteriocins are antimicrobial peptides synthesized by some bacterial strains that have a narrow spectrum of activity, affecting a select population of bacteria without disrupting the greater commensal microbiota [21,22]. With increased prevalence of antibiotic resistance in *S. suis* and increased pressure to reduce antibiotic usage in animal agriculture, bacteriocins are of interest as a novel intervention strategy against bacterial pathogens in swine. *S. suis* 90–1330 is an avirulent *S. suis* serotype 2 strain that produces a bacteriocin of the lantibiotic class [16,23]. The bacteriocin, named suicin, shows efficacy against 15 pathogenic strains of *S. suis* serotype 2 (sequence type 1 and 25) and is chromosomally located, making it a strong candidate for further investigation [16,24].

In this work, we evaluated the use of *S. suis* strain 90–1330 as an intranasal vaccine. We hypothesized the strain would colonize the upper respiratory tract of inoculated animals and reduce disease following subsequent challenge with a virulent *S. suis* serotype 2 strain, due to the presence of suicin.

## Materials and methods

### Isolates and culture conditions

The *S. suis* strain 90–1330 was isolated from the lung of a pig and has been previously verified to produce a bacteriocin that can inhibit the growth of other *S. suis* isolates [16,25]. The virulent serotype 2 strain P1/7 was used as the challenge strain. Other *S. suis* isolates used as bacteriocin controls include 89–1591, MGGUS13, and DAT229 [23,25]. Isolates were routinely grown on blood agar plates (BD Biosciences, Franklin Lakes, NJ) incubated at 37ºC and 5% CO_2_ unless otherwise specified.

### Construction of a Δ*suicin* mutant

A precise, in-frame deletion of the gene encoding the suicin in wild-type strain 90–1030 (gene locus tag AN924_RS11115, GenBank Accession NZ_CP012731 [24]) was constructed by using splicing-by-overlap-extension PCR [26]. Oligonucleotide primers (ThermoFisher Scientific, Mississauga, Canada) used for the construction of the deletion allele are listed in S1 Table. Appropriate deletion alleles were cloned into plasmid pCR2.1 (ThermoFisher), extracted with BamHI and PstI and recloned into the thermosensitive *E. coli-S. suis* shuttle vector pSET4s [27] digested with the same enzymes. Electroporation of *S. suis* and procedures for isolation of mutants were those described previously [28]. Deletion of gene AN924_RS11115 in the resulting mutant strain 90–1330Δ*suicin* was confirmed by PCR and sequencing analysis. Genomic DNA was prepared by the guanidium thiocyanate method [29]. Minipreparations of recombinant plasmids and transformation of *E. coli* were performed by standard procedures [30]. Restriction enzymes and DNA-modifying enzymes were ThermoFisher. PCR reactions were carried out with the iProof proofreading DNA polymerase (BioRad Laboratories, Hercules, CA).

### Plate diffusion assay for bacteriocin production

Bacteriocin production by the wild-type strain and its suicin-negative mutant derivative were assessed by plate diffusion as previously described [16]. Briefly, overnight cultures of both wild-type strain 90–1030, its isogenic mutant 90–1330*Δsuicin*, as well as *S. suis* positive and negative control for suicin production (strains MGGUS13 and DAT229, respectively) were spotted (2 μL) onto Todd-Hewitt agar (THA; BBL Microbiology Systems, Mississauga, Canada) plates and incubated at 37°C for 24 hours. The plates were then overlaid with THB soft agar (0.75% [wt/vol]) that had been inoculated (750 μL in 7 mL) with a 24-hour culture of indicator *S. suis* strains and further incubated at 37°C for 24 hours. The zones of inhibition were measured from the edge of the growth of *S. suis* to the margin of the inhibitory zone.

### Serum antibody assessment

Serum antibody titers were evaluated by enzyme linked immunosorbent assay (ELISA), as previously described [31]. Plates were coated with 0.5 µg/mL *S. suis* strain P1/7 or strain 90–1330 whole cell sonicate in 100 mM carbonate-bicarbonate buffer. Serum samples were assessed in duplicate with two-fold serial dilutions and detected with horse radish peroxidase conjugated goat anti-pig immunoglobulin (SeraCare, Milford, MA). The reaction was developed for 10 minutes with tetramethylbenzidine (TMB) substrate (Life Technologies, Carlsbad, CA) and stopped with 2N H_2_SO_4_. Titers were determined by modeling a nonlinear function using the log_10_ dilution and log (agonist) versus response variable slope four-parameter logistic model with cutoff values set at two times the average value of the gnotobiotic pig serum control.

### Opsonophagocytosis assay (OPA)

The OPA was performed as previously described [11]. Briefly, whole blood (as a source of phagocytic cells) was collected by vacutainer from 4- to 8-week-old piglets (in the absence of significant levels of maternal antibodies) in sodium heparin tubes (Becton Dickinson, Franklin Lakes, NJ). Piglets came from a high health status herd without *S. suis* endemic disease. Bacterial cultures of strain 90–1330 were washed and resuspended in complete cell culture medium (RPMI 1640 supplemented with 5% heat-inactivated fetal bovine serum, 10 mM HEPES, 2 mM L-glutamine and 50 μM 2-mercaptoethanol; Invitrogen) at a concentration of 6.25x10^5^ CFU/mL. *S. suis* concentration was verified by plating on THB agar (THA). The *S. suis* suspension was combined with whole blood containing approximately 1x10^8^ leukocytes/mL to obtain and multiplicity of infection (MOI) of 0.01. Control serum or serum from study animals was added to a concentration of 20% (v/v) to obtain a final volume of 200 µ L. Negative control serum was obtained from naïve pigs and adsorbed against *S. suis* resulting in a negative ELISA value. Positive serum was a pooled sample from sows with high positive ELISA values. The test was set up in microcentrifuge tubes and the tube tops were pierced with a sterile needle. Samples were incubated for 2 hours at 37ºC with 5% CO_2_. Tubes were mixed by tapping every 15 minutes to facilitate increased bacterial-leukocyte interactions. After incubation, bacterial survival was determined by plating serial dilutions on THA plates. Percent killing was determined using the formula: % bacteria killed = [1 - (CFU from test serum/ CFU from adsorbed serum)] x 100.

### Animal experiments

All animal studies were approved by the USDA-ARS National Animal Disease Center’s Institutional Animal Care and Use Committee under protocol number ARS-23–1091 and ARS-23–1097. All cesarean derived, colostrum deprived (CDCD) pigs were derived and raised at the National Animal Disease Center. Animal study designs are depicted in S1 Fig. Nasal samples taken prior to the start of study were plated on blood agar to confirm the absence of *S. suis* in CDCD animals. Animals challenged with *S. suis* were evaluated twice daily. If clinical signs were observed but not severe enough to warrant euthanasia, a third daily observation was made overnight. Animals were euthanized by pentobarbital overdose immediately when humane endpoints were met. Euthanasia criteria included: lethargy/depression resulting in failure to rise upon pen entry, non-weight bearing lameness for 48 hours, neurologic signs resulting in recumbency, seizures, cyanosis or labored breathing, and loss of body condition.

### Animal study 1

To evaluate the virulence of *S. suis* strain 90–1330 in CDCD pigs and evaluate the impact of dose on colonization, pigs were inoculated with 90–1330 as a low dose (10^6^) or a high dose (10^9^). Twenty-six seven-week-old CDCD pigs were divided into three groups: group 1 pigs (n = 9) were inoculated with 9x10^6^ colony forming units (CFU)/mL of 90–1330, group 2 pigs (n = 9) were inoculated with 10^9^ CFU/mL of 90–1330, and group 3 pigs (n = 8) were inoculated with 9x10^8^ CFU/mL of the virulent P1/7 strain as a control for *S. suis* susceptibility. Pigs were inoculated intranasally and monitored for 21 days. Colonization with 90–1330 was evaluated on days 1, 3, 7, 14, and 21. Nasal swabs were taken, and serial dilutions were plated on blood agar plates. *S. suis* was quantified by CFU counts. Serum was collected from animals on day 0 and 21 to evaluate antibody titers. Pigs that developed severe disease were euthanized and necropsied. Lesions were noted and the following samples were collected to evaluate the distribution of *S. suis*: nasal swab, tonsil swab, serum, joint fluid, serosal swab, cerebrospinal fluid (CSF), and bronchoalveolar lavage fluid (BALF). To evaluate for protective effect of colonization, after 21 days, surviving pigs in groups 1 and 2 were challenged intranasally with 3x10^9^ CFU/mL of P1/7. Pigs developing clinical signs of disease were euthanized and necropsied as described. Surviving pigs were euthanized 14 days post-challenge with the P1/7 strain and assessed for systemic disease.

### Animal study 2

To evaluate whether protection 21 days after exposure was associated with bacteriocin production, 21 seven-week-old CDCD pigs were divided into two groups: group 1 pigs (n = 11) were inoculated with 3x10^8^ CFU/mL of 90–1330Δ*suicin* and group 2 pigs (n = 10) were sham inoculated with PBS. Nasal swabs were collected on days 0, 1, 3, 7, 14, and 21 to evaluate colonization and serum was collected on days 0 and 21 to assess antibody titers. Similar to animal study 1, animals in both groups were challenged with 3x10^9^ CFU/mL of P1/7 strain 21 days post-inoculation. Animals were evaluated and assessed for systemic disease as described above. Two weeks post-challenge, surviving animals were euthanized and evaluated for systemic signs of disease.

### Animal study 3

To evaluate the impact of the bacteriocin on disease after challenge with the virulent strain P1/7 prior to the development of antibody titers, 28 seven-week-old CDCD pigs were divided into three groups: group 1 pigs (n = 10) were inoculated with 4x10^8^ CFU/mL of 90–1330, group 2 pigs (n = 9) were inoculated with 1x10^9^ CFU/mL of 90–1330Δ*suicin*, and group 3 (n = 9) were sham inoculated with PBS. Nasal swabs were collected on days 0, 1, and 3 to assess colonization and serum was collected on days 0 and 3 to evaluate antibody titers. On day 3 post inoculation, animals were challenged with 2x10^9^ CFU/mL of P1/7. Animals were evaluated as described and euthanized if disease became severe. Surviving animals were euthanized 14 days post-challenge.

### Statistical analysis

Statistical analysis was performed using GraphPad Prism software (GraphPad, La Jolla, CA). Differences were considered significant at the P < 0.05 level. Survival was assessed using the product limit method of Kaplan and Meier and survival curves were compared using the log-rank test. Antibody titers were converted to a log_10_ scale for comparison. Differences between groups and within groups at different timepoints were compared using a two-way ANOVA. Percent killing by OPA was compared using a two-way ANOVA (animal study 1) and T tests (animal study 2 and study comparison).

## Results

### Mutant generation and verification

We generated a mutant strain devoid of suicin production by inactivation of gene AN924_RS11115 by precise, in frame, allelic replacement in parental strain 90–1330. The deletion was confirmed by PCR and Sanger sequencing. The resulting mutant strain, 90–1330*Δsuicin* exhibited a growth kinetic similar to that of the wild-type parental strain upon cultivation in standard laboratory media (S2 Fig). To verify whether the 90–1330*Δsuicin* was impaired in suicin production, we evaluated the capacity of the mutant to inhibit growth of different *S. suis* strains in a plate diffusion assay. As shown in Table 1, while the wild-type strain and the positive control strain (MGGUS13) produced inhibitory zones of various sizes (3–4 mm) against the strains tested, the mutant strain 90–1330Δ*suicin* behaved like the negative control strain (DAT229) and did not inhibit growth of these strains, thus confirming that the genetic exchange resulted in abolishment of suicin production.

**Table 1:** Verification of the loss of suicin production. Inhibition of the growth of different test *S. suis* strains sensitive to suicin by strain 90-1330, its derivative mutant 90-1330Δ*suicin*, strain MGGUS13 (known to produce suicin), and strain DAT229 (known to not produce suicin). The 90-1330Δ*suicin* mutant lost its ability to produce suicin, as evidenced by its lack of inhibition of growth of susceptible strains P1/7 and 89-1591.

	Test strain (zone of inhibition in mm)		
	90-1330	P1/7	89-1591
90-1330 (+)	1	4	3
90-1330Δ*suicin* (-)	0	0.5	0
MGGUS13 (+)	1	4	3
DAT229 (-)	0	0	0

### Animal study 1

To ensure that *S. suis* strain 90–1330 was avirulent in the CDCD pig model, pigs were inoculated with a high and low dose of the 90–1330 strain. The P1/7 strain of known virulence was used as a positive control to ensure susceptibility of the animals to *S. suis*. Animals in groups 1 and 2 were inoculated with *S. suis* strain 90–1330. There were no animals in either group that displayed clinical signs of *S. suis* disease. Animals in group 3 were challenged with *S. suis* strain P1/7. Six of the eight animals in this group developed clinical signs of disease, including anorexia, lethargy, neurologic signs, and lameness and were euthanized for humane reasons.

**Fig 1:**
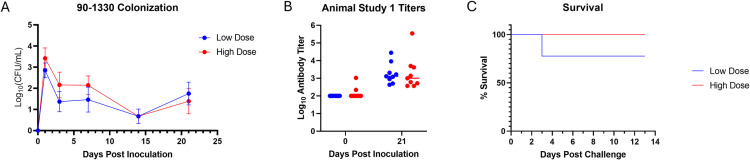
Colonization, serum antibody titers, and survival for animal study 1. (A) Colonization with *S. suis* strain 90-1330 was enumerated by plating dilutions on blood agar plates. *S. suis* was highest day 1 post-inoculation and dropped on subsequent samplings. Pigs in the high dose group had higher *S. suis* colonization early following inoculation, but levels were comparable at later time points. Though numerical differences were observed, the differences were not statistical at any time point. (B) Serum antibody titers were assessed by ELISA. Serum antibody titers were higher for both groups after exposure to 90-1330 (day 21, P < 0.01). There was no difference in titer between the high and low dose groups. (C) Survival of animals after challenge with *S. suis* P1/7 was comparable between the low dose group (7/9) and high dose group (9/9).

Nasal colonization with the strain 90–1330 was tracked for groups 1 and 2 on days 1, 3, 7, 14 and 21 post-inoculation (Fig 1-A). Colonization was not statistically different between group 1 and 2 at any time point; however, there tended to be a higher colonization level in the high dose group (group 2) than the low dose group (group 1) for the first week following inoculation. Colonization was highest for both groups on day 1 post-inoculation, decreased by day 3 post-inoculation, and remained at similar levels from day 3–21 post-inoculation.

Serum antibody titers were evaluated for groups 1 and 2 prior to inoculation (day 0) and on day 21 post-inoculation to assess immune stimulation by strain 90–1330 (Fig 1-B). Titers were similar between the low and high dose groups (P = 1.0) and titers rose from day 0 to day 21 in both groups (P < 0.01).

On day 21, pigs in groups 1 and 2 were challenged with *S. suis* strain P1/7 to assess whether prior inoculation with the bacteriocin producing strain 90–1330 could reduce the development of clinical disease following challenge with a virulent strain (Fig 1-C). After challenge, two pigs in the low dose group developed clinical signs of *S. suis* and were euthanized. No pigs in the high dose group developed clinical disease. When compared to the animals in group 3 challenged with the strain P1/7 on day 0 of the study, there was prolonged survival and reduced mortality in pigs receiving the high dose of 90–1330 (group 2); however, the differences in median survival time and survival did not reach the statistical threshold for the low dose group (group 1). The results of animal study 1 showed 90–1330 was avirulent in a CDCD model and stimulated protection against virulent *S. suis* challenge 21 days after inoculation.

### Animal study 2

**Fig 2:**
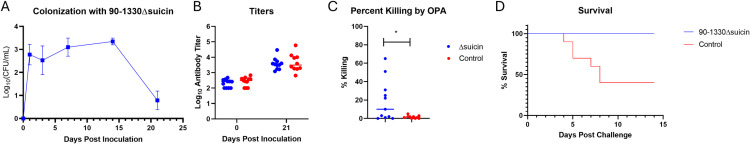
Colonization, serum antibody titers, OPA killing, and survival for animal study 2. (A) Colonization with *Streptococcus suis* 90-1330Δsuicin was enumerated by dilution plating. Colonization with 90-1330Δ*suicin* was similar to colonization with wild-type 90-1330 (from animal study 1) over the study period. (B) Serum antibody titers were measured by ELISA. Antibody titers rose for both groups over the study period. Animals inoculated with 90-1330Δ*suicin* had similar titers to control animals on day 0 and 21 post-inoculation. (C) Functional antibody was assessed by opsonophagocytosis assay (OPA). A higher percent killing was seen for animals inoculated with 90-1330Δ*suicin* than control animals on day 21 post-inoculation (P = 0.02). (D) Survival of animals inoculated with 90-1330Δ*suicin* was higher than that of the control group (P = 0.0039). No animals inoculated with 90-1330Δ*suicin* showed clinical signs of disease following challenge with *S. suis* P1/7.

Since the results of animal study 1 indicated prior inoculation with 90–1330 provided protection against subsequent virulent challenge, a second study was performed with the mutant strain lacking bacteriocin production (90–1330Δ*suicin*) to determine if bacteriocin production contributed to the protective response. Colonization with 90–1330Δ*suicin* was evaluated on days 1, 3, 7, 14, and 21 post-inoculation (Fig 2-A). Colonization with 90–1330Δ*suicin* averaged 10^2^–10^3^ CFU/mL day 1–14 post-inoculation and decreased by day 21. Colonization was similar between animals inoculated with wild type 90–1330 (animal study 1) and 90–1330Δ*suicin* days 1, 3, 7, and 21 post-inoculation. Differences in colonization between wild type 90–1330 and 90–1330Δ*suicin* were noted on day 14 post-inoculation.

Serum antibody titers were evaluated on days 0 and 21. Titers were similar between 90–1330Δ*suicin* inoculated animals and control animals on day 0 and day 21 post-inoculation (Fig 2-B); however, when antibody function was assessed (OPA test), 90–1330Δ*suicin* inoculated animals showed greater *S. suis* killing than control animals (P = 0.02; Fig 2-C). When directly compared titers were similar between 90–1330Δ*suicin* inoculated animals from animal study 2 and animals inoculated with wild type 90–1330 from animal study 1 on day 21 post-inoculation (P = 0.23).

On day 21 post-inoculation, animals in groups 1 and 2 were challenged with *S. suis* P1/7 strain (Fig 2-D). No animals inoculated with 90–1330Δ*suicin* developed *S. suis* disease (0/11), while 6/10 animals in the control group developed *S. suis* disease and had to be euthanized (P = 0.0039). Survival of pigs inoculated with 90–1330Δ*suicin* in animal study 2 was similar to pigs inoculated with 90–1330 in animal study 1. The data from animal study 2 indicates that production of suicin by 90–1330 was not essential for protection against challenge with virulent *S. suis.*

### Animal study 3

**Fig 3:**
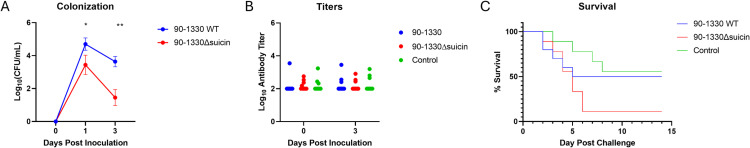
*S. suis* colonization, serum antibody titers, and survival for animal study 3. (A) Colonization was assessed by dilution plating. Strain 90–1330 wild-type colonized at higher concentrations than 90–1330Δsuicin on D1 and D3. Statistical differences are indicated by the asterisks (* P < 0.05; ** P < 0.01). (B) Serum antibody titers were evaluated by ELISA. Similar titers were seen in all groups on D0 and the day of challenge with *S. suis* strain P1/7 (D3). (C) Survival following challenge with *S. suis* strain P1/7 was similar between groups (P = 0.10).

Since both 90–1330 and 90–1330Δ*suicin* strains protected against virulent challenge 21 days post-inoculation and increases in antibody titer were noted in animal studies 1 and 2 which could have contributed to protection, a third animal study was completed to determine if the production of suicin could prevent disease following virulent challenge in the absence of antibody titers. In animal study 3, animals were challenged with virulent *S. suis* 3 days after inoculation with 90–1330 or 90–1330Δ*suicin*, prior to the development of a humoral response. Colonization with 90–1330 and 90–1330Δ*suicin* was assessed on days 1 and 3 post-inoculation (Fig 3-A). Colonization was significantly higher for pigs inoculated with 90–1330 than the mutant strain on both day 1 and 3. Serum antibody titers were comparable among groups at the time of challenge with *S. suis* strain P1/7 (Fig 3-B). Clinical signs were noted in 7/10 animals inoculated with the strain 90–1330, 9/9 animals inoculated with the mutant strain 90–1330Δ*suicin*, and 6/9 animals inoculated with PBS (control group). Disease became severe and required euthanasia in 5/10 animals inoculated with strain 90–1330, 8/9 animals inoculated with 90–1330Δ*suicin*, and 4/9 animals inoculated with PBS (Fig 3-C). Survival following challenge with *S. suis* P1/7 strain was not statistically different among groups (P = 0.10). The results of animal study 3 suggest protection provided by 90–1330 takes time to develop and is immune mediated.

## Discussion

*S. suis* causes significant economic losses for the swine industry worldwide. Prevention of systemic disease with *S. suis* has been challenging, due to difficulty developing broadly protective vaccines and the incomplete protection seen with bacterin vaccines [9,10]. Identification of alternative or novel vaccines and vaccine platforms is critical to reducing antibiotic use and losses due to *S. suis*. In this study, we evaluated the use of a bacteriocin producing strain of *S. suis* (90–1330) as an avirulent, intranasal vaccine to protect against challenge with a virulent *S. suis* isolate. This novel approach would provide a multi-layered defense against *S. suis* by stimulating the immune system and providing colonization resistance through bacteriocin production. We assessed colonization, antibody titers, functional antibody, and compared survival between animals inoculated with 90–1330 wild-type or 90–1330Δ*suicin* to understand the role of suicin and evaluated protection by challenging animals 21 days and three days after inoculation.

Colonization was similar between 90–1330 wild-type (high dose, Animal Study 1) and the mutant strain (Animal Study 2) over the 21 days post-inoculation. In both studies, animals were protected from systemic disease with *S. suis* strain P1/7 when challenged 21 days following exposure to 90–1330 wild-type or 90–1330Δ*suicin*. Systemic protection against *S. suis* has been seen previously with intramuscular or intravenous inoculation of live avirulent strains, including 90–1330 [32–34]; however, this is the first report of protection following intranasal inoculation. Interestingly, in this study protection was seen after a single intranasal inoculation of 90–1330, while previous work observed protection after two or three intramuscular inoculations [32]. Because protection was seen with animals inoculated with the wild-type and Δ*suicin* and an increase in systemic antibody titer and opsonic activity of antibody was observed in inoculated animals, we speculated the protection was due to humoral immunity rather than suicin production, although competitive inhibition could not be excluded.

To determine the impact of suicin production on preventing systemic disease and evaluate whether competitive inhibition was contributing to protection against challenge, we assessed protection against virulent challenge prior to the development of a systemic antibody response (three days post-inoculation). Animals inoculated with 90–1330 wild-type or 90–1330Δ*suicin* were not protected against systemic disease when challenged with *S. suis* strain P1/7 three days after inoculation. This suggests that antibodies and increased opsonic activity produced following inoculation with 90–1330 and 90–1330Δ*suicin* were likely the main contributor to protection in animals challenged 21 days after inoculation. Although Zhu et al previously found that bacteriocins can improve survival against virulent *S. suis* challenge in a mouse model [19], in this study, suicin did not appear to play a role in protection. However, the differences may have been due to the differences in bacteriocins (nisin compared to suicin), the dosage of bacteriocin, the model (mice compared to swine), or other factors. The usage of 90–1330 described in this study is intended for disease prevention rather than treatment.

Interestingly, we noted differences in colonization between 90–1330 wild-type and 90–1330Δ*suicin* in Animal Study 3, though levels were statistically similar when comparing Animal Study 1 and 2. Each animal study described was completed using a different group of animals, which were from different sows and raised at different times. Differences in genetics and microbiota development could contribute to differences in colonization between the studies; however, Animal Study 3 is the only direct comparison between 90–1330 wild-type and 90–1330Δ*suicin*, which indicates 90–1330 wild-type may have a competitive colonization advantage over 90–1330Δ*suicin*. This is not unexpected, as suicin may play a role in eliminating closely related bacteria that may occupy the same niche as *S. suis*. This competitive advantage has been noted for other bacterial species, including other Streptococci, that produce bacteriocins [35,36].

Although vaccination with 90–1330 wild-type and 90–1330Δ*suicin* did not protect animals from challenge three days post inoculation in this study, 90–1330 still has good potential as a live vaccine. In this study, animals were challenged with 10^9^ CFU/mL of virulent *S. suis*, which may have overwhelmed suicin production by 90–1330. However, under field conditions with lower exposure doses, the production of suicin may be better able to prevent systemic infection. To determine whether suicin production would be effective under field-like conditions, protection following challenge with lower inoculum dose or using a transmission model should be assessed. The impact of inoculation with 90–1330 on the microbiome is unknown, and prior to its use in a field setting, further work should investigate if suicin significantly alters the microbiota of conventional animals. Importantly, 90–1330 is an avirulent isolate and was able to stimulate a protective antibody response with a single inoculation within 21 days of exposure, which makes it a valuable candidate for vaccine development.

## Supporting information

S1 Table Table S1: Primers for constructing S. suis 90-1330∆suicin mutant. (XLSX)

S1 Fig Animal study designs. The study designs for the three animal studies are depicted. Nasal swabs are indicated by an orange arrow, blood draws are indicated by a syringe, and inoculation is indicated by the bacterial icon. Pigs were inoculated on day 0 with the aviurlent wild-type or mutant strain (∆*suicin*), represented by the green bacterial icon. Pigs were euthanized 14 days post-challenge with the pathogenic *S. suis* strain P1/7 (red bacterial icon).
(TIF)

S2 Fig Growth curve of *Streptococcus suis* strain 90–1330 and its isogenic mutant 90–1330Δ*suicin.* Both parental and mutant strains showed similar growth kinetics in Todd-Hewitt broth for up to 24h.
(TIF)
