# Supplementary figures and images for: Protection induced in pigs previously infected by the non-virulent strain 1330 of Streptococcus suis serotype 2 is not due to the secretion of the bacteriocin suicin
Source: PLoS One. 2025 May 29;20(5):e0323370. doi: 10.1371/journal.pone.0323370 (PMC12122026; doi:10.1371/journal.pone.0323370)

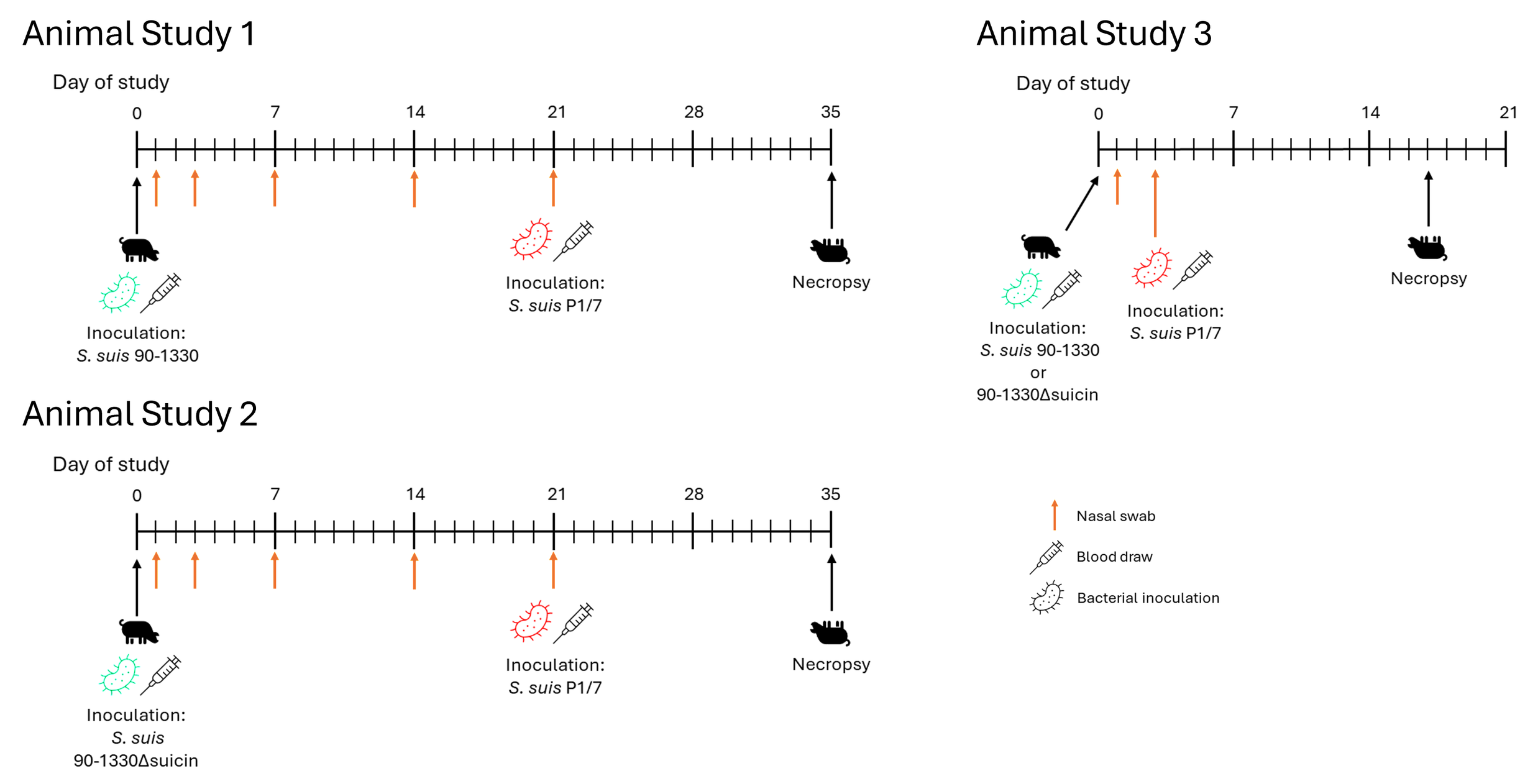

Supplement: S1 Fig — The study designs for the three animal studies are depicted. Nasal swabs are indicated by an orange arrow, blood draws are indicated by a syringe, and inoculation is indicated by the bacterial icon. Pigs were inoculated on day 0 with the aviurlent wild-type or mutant strain (∆suicin), represented by the green bacterial icon. Pigs were euthanized 14 days post-challenge with the pathogenic S. suis strain P1/7 (red bacterial icon). (TIF) [file pone.0323370.s001.tif]

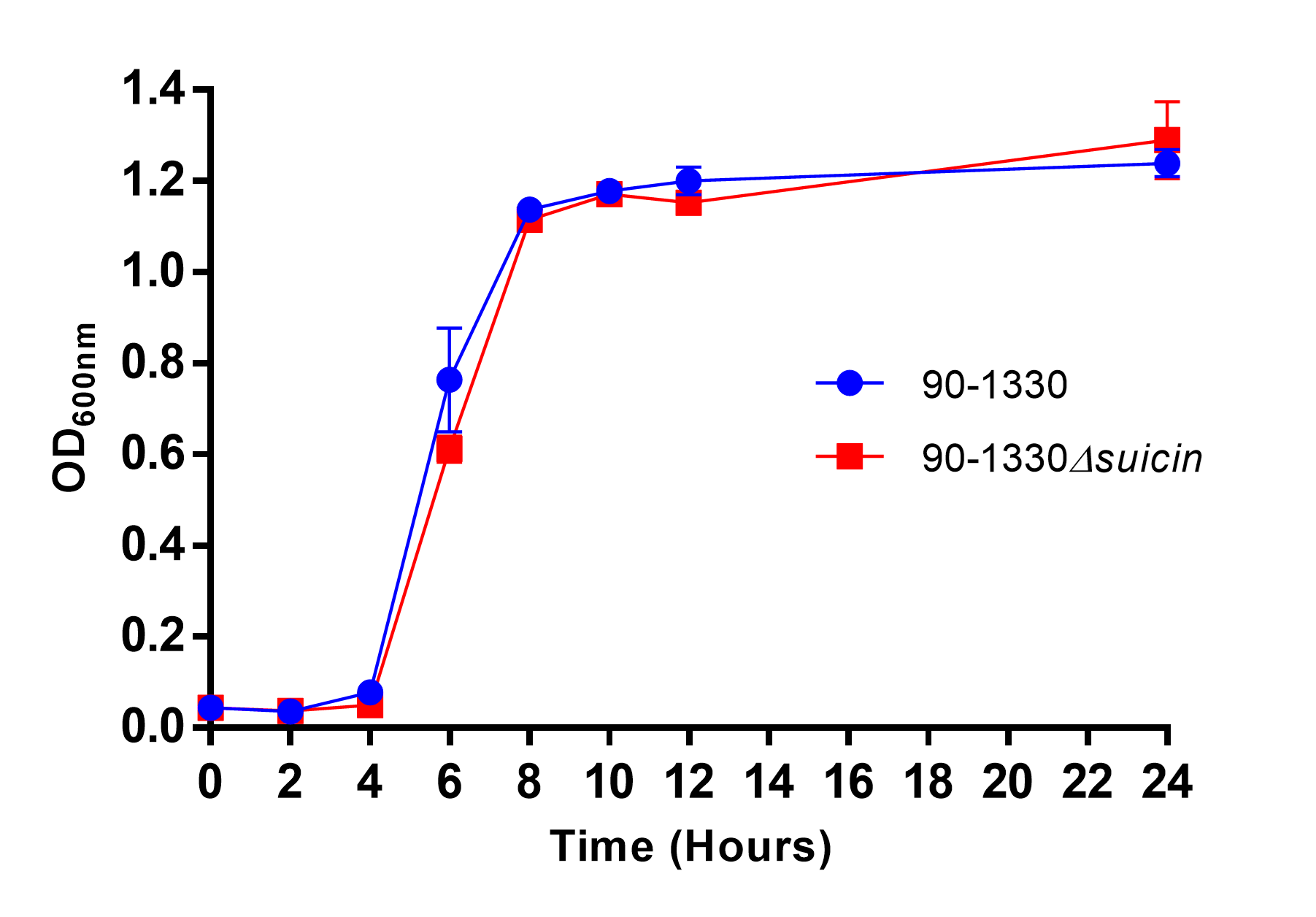

Supplement: S2 Fig — Both parental and mutant strains showed similar growth kinetics in Todd-Hewitt broth for up to 24h. (TIF) [file pone.0323370.s002.tif]
